# The Multifaceted Role of Osteopontin in Prostate Pathologies

**DOI:** 10.3390/biomedicines11112895

**Published:** 2023-10-26

**Authors:** Samara V. Silver, Petra Popovics

**Affiliations:** 1 Department of Microbiology and Molecular Cell Biology, Eastern Virginia Medical School, Norfolk, VA 23507, USA; silversv@evms.edu; 2 Leroy T. Canoles Jr. Cancer Research Center, Eastern Virginia Medical School, Norfolk, VA 23507, USA

**Keywords:** prostatitis, benign prostatic hyperplasia, prostate cancer, foam cells

## Abstract

The prostate gland, located beneath the bladder and surrounding the proximal urethra in men, plays a vital role in reproductive physiology and sexual health. Despite its importance, the prostate is vulnerable to various pathologies, including prostatitis, benign prostatic hyperplasia (BPH) and prostate cancer (PCa). Osteopontin (OPN), a versatile protein involved in wound healing, inflammatory responses, and fibrotic diseases, has been implicated in all three prostate conditions. The role of OPN in prostatic pathophysiology, affecting both benign and malignant prostate conditions, is significant. Current evidence strongly suggests that OPN is expressed at a higher level in prostate cancer and promotes tumor progression and aggressiveness. Conversely, OPN is primarily secreted by macrophages and foam cells in benign prostate conditions and provokes inflammation and fibrosis. This review discusses the accumulating evidence on the role of OPN in prostatic diseases, cellular sources, and potential roles while also highlighting areas for future investigations.

## 1. Introduction

The prostate gland, situated below the bladder and around the proximal urethra in men, is pivotal for sexual function. It secretes a substantial fraction of the seminal fluid, particularly prostate-specific antigen (PSA). PSA, an alkaline phosphatase, aids in liquifying the ejaculate, facilitating sperm mobility and ensuring an optimal pH for spermatozoa survival within the vaginal environment [1]. Moreover, the autonomic smooth muscle fibers of the prostate contribute to semen expulsion during ejaculation, while its rich sensory innervation has implications for sexual pleasure [2].

Despite its crucial role in sexual health, the prostate is susceptible to multiple pathologies throughout the life of a man. Prostatitis is the most common urological problem in men below the age of 50. It is defined based on urological pain derived from the prostate due to infection or abacterial inflammation, but sometimes the causes are unknown [3]. Benign prostatic hyperplasia (BPH) results from a complex non-malignant pathological change in the prostate, leading to urethral compression and lower urinary tract symptoms (LUTS), including increased urinary frequency, nocturia, and urinary retention [4]. The pathology of male BPH/LUTS includes non-malignant growth driven by proliferation or resistance to apoptosis, as well as inflammation, smooth muscle dysfunction, and fibrosis [5,6]. Moderate to severe LUTS affect 33% of men in their 60s, substantially reducing their quality of life [6]. Prostate cancer is the most diagnosed and second deadliest non-cutaneous malignancy in men in the US [7]. Its etiology, while multifactorial, has strong correlations with age, genetics, inflammation, diet, and obesity [8]. While androgen-deprivation therapy (ADT) proves effective for the majority of patients with prostate cancer (PCa), a subset might progress to castration-resistant prostate cancer (CRPC), a form characterized by heightened aggressiveness and potential lethality, within a few years of treatment [9].

Men who develop BPH exhibit a higher incidence of prostate cancer [10]; however, the prevailing consensus in the medical community is that BPH does not progress to malignancy. Their separate etiologies are underlined by a distinct zonal localization in the prostate; BPH develops exclusively in the transition zone, whereas PCa is primarily localized in the peripheral zone [11,12]. Chronic inflammation can manifest in both the transition and peripheral zones of the prostate, reinforcing its association with the development of both BPH and PCa [12].

Osteopontin (OPN), a multifunctional protein involved in various physiological processes, notably in wound healing, inflammatory responses, particularly those of autoimmune nature, and fibrotic diseases [13,14,15,16,17,18,19], has been implicated in all three major prostate pathologies. This review seeks to integrate the available evidence on the role of OPN in prostate pathologies with insights from its involvement in other diseases while also speculating its potential cellular functions within the prostate.

## 2. Osteopontin: Structure, Splice Variants, and Roles in Non-Prostatic Tissues

OPN, when secreted in injured tissues, modulates immune cell migration, leverages its adhesive capabilities to retain immune cells [20], and orchestrates Th1-cell mediated immune response [21]. The sustained secretion of OPN is intricately associated with the onset of chronic inflammatory and autoimmune disorders, including but not limited to Crohn’s disease [13], rheumatoid arthritis [14], and inflammatory bowel disease [15]. Moreover, the implications of OPN are profoundly evident in a spectrum of fibrotic diseases. It delays the resolution of thioacetamide-induced liver fibrosis in murine models, attributed partially to its potent stimulation of collagen-I deposition [16]. Furthermore, analogous impacts of OPN have been demonstrated in studies exploring idiopathic pulmonary fibrosis [17], diabetes-induced interstitial fibrosis of the kidney [18], and cardiac fibrosis [19], underscoring its pervasive role across varying fibrotic conditions.

OPN is a sialic acid-rich protein, originally identified in the bone extracellular matrix, and can exist as an immobilized or as a soluble protein [22,23,24]. There are several synonyms for OPN, including early T-lymphocyte activation and bone sialoprotein, and it is encoded by the gene *SPP1* (secreted phosphoprotein I). OPN belongs to the SIBLING (small integrin-binding ligand N-linked glycoprotein) protein family and is a secreted, heavily glycosylated, and phosphorylated protein. OPN is also an intrinsically disordered protein, denoting that it lacks a defined tertiary structure and potentially has multiple structural states allowing its interaction with various binding partners [25]. Osteopontin has one of the highest densities of phosphorylation sites amongst extracellular matrix proteins [26] mainly generated by Fam20C [27]. Phosphorylation affects its binding to hydroxyapatite, thereby modulating its activity on bone growth, remodeling, and calcification [28,29]. Glycosylation affects phosphorylation level and adhesive properties of OPN and its binding to integrins [13]. In addition, transglutaminase-mediated oligomerization of OPN increases cell adhesion [30]. Proteolytic cleavage also contributes to the diversity of actions of OPN; processing by thrombin regulates receptor specificity and can release a c-terminal chemotactic protein fragment [31,32]. OPN is also cleaved by matrix metalloproteinases, plasmin, cathepsin D, and caspase 8, providing increased activity or deactivation [33,34,35].

Five main splice variants of OPN exist. OPNa represents the full-length splice variant of OPN. Conversely, OPN-b is characterized by the absence of exon 5, and OPNc is devoid of exon 4. A further variant, OPN-4, lacks both exons 4 and 5 [36]. Additionally, OPN-5 uniquely includes an extra exon, which is formulated by retaining a segment of intron 3, whereas it lacks the signal sequence required for its loading to secretory vesicles, rendering it intracellular [37]. Subsequently, four variants of OPN-5 (OPN-5b, OPN-5c, OPN-5d, and OPN-5e) have also been proposed [38], and it has been suggested that they are secreted, although with less probability than other variants [39]. Splice variants may differ in function; OPN-b regulates cell migration and adhesion to laminin, whereas OPNc has decreased activity on migration [40]. In addition, OPN-5 has been proposed to confer important intrinsic roles regulating receptor function and acting as a scaffold protein in immune cells [41].

OPN binds to and activates several integrins as well as the CD44 receptor. The Arg-Gly-Asp (RGD) sequence that is positioned on exon 6 interacts with αvβ1, αvβ3, and αvβ5 [32], whereas the alternative integrin binding site exposed after thrombin cleavage (SVVYGLR) binds to α9β1, α4β1, and α4β7 integrins [42]. Polymerization also creates a novel α9β1 binding site that drives neutrophil chemotaxis [43]. Binding of OPN via the CD44 receptor regulates cell attachment and chemotaxis [44]. Strategies to prevent OPN-receptor binding have been largely explored due to the involvement of OPN in various malignancies and inflammatory conditions. These include RNA or DNA aptamers that prevent the binding of OPN to multiple receptors (CD44 and αvβ3 integrin) [45].

## 3. Benign Prostate Disease

Prostatitis, a multifaceted inflammatory condition of the prostate gland, is taxonomically classified into four distinct categories: acute bacterial prostatitis (Category I), chronic bacterial prostatitis (Category II), chronic prostatitis or chronic pelvic pain syndrome (Category III), and asymptomatic inflammatory prostatitis (Category IV) [3]. Histological characterization of prostatitis is scarce due to the limited availability of tissues because the prostate gland is seldom excised solely based on this condition. In contrast, BPH is known to manifest a rich histological heterogeneity, characterized by nodular tissue proliferation. These nodules predominantly comprise stromal or glandular elements, but fibrotic components are also discernible. Intriguingly, an overwhelming majority of BPH tissue samples reveal evidence of chronic inflammation and are rich in macrophages and T and B lymphocytes [46]. Such consistent observations have led to the postulation that prostatic inflammation may be one of the pivotal etiological factors driving the pathogenesis of BPH [47,48]. This hypothesis is supported by numerous animal studies in which inflammatory prostatitis was induced, revealing pathological changes consistent with BPH, including cellular proliferation and fibrosis [49,50,51]. Given these findings, prostatitis and BPH will be jointly examined in this section.

### 3.1. Benign Prostatic Hyperplasia Tissue

BPH tissues are frequently employed as control samples for prostate cancer studies due to their more accessible nature compared to normal prostate tissues. Nevertheless, these studies commonly uncover significant insights regarding the pathogenesis of BPH. A study by Thalmann et al. highlighted a similar expression of OPN mRNA in both prostate cancer and BPH tissues [52]. Intriguingly, BPH patients exhibited elevated serum levels of OPN in comparison to age-matched healthy males. These increased OPN levels correlated with a surge in MMP-9, a molecule recognized to be activated by OPN [53]. Additionally, the prevalence of serum OPN antibodies, suggesting an autoimmune response against OPN, was found to be heightened in BPH patients relative to healthy donors [54].

To deepen our understanding on the role of OPN in BPH pathogenesis, our team evaluated the tissue levels and expression patterns of OPN in tissues from patients undergoing surgery for LUTS (surgical BPH) compared to those from patients with low-risk, small-volume prostate cancer exhibiting incidental BPH histology, representing an earlier disease stage [55]. This analysis revealed that surgical samples contain elevated OPN levels, especially notable in those patients administered a combination of α-blockers and 5-α reductase inhibitors. This observation suggests a correlation with disease progression [55]. Furthermore, these patients receiving the combination treatment display the most substantial collagen accumulation in the prostate [56], indicating fibrosis. We also confirmed the localization of OPN in glandular cells (with occasional heightened expression in basal cells), in the stromal compartment, in endothelial cells, and in inflammatory infiltrates [55]. Characterization of OPN in prostate cell lines identified main molecular masses of 42, 52, and 70 kDa as well as the 32 kDa cleaved peptide in both benign and PCa cells, whereas the M0-differentiated THP-1 macrophages show a switch to unique OPN variants of 48 and 68 kDa. In human tissues and stromal cells, the dominant OPN variant is the 32 kD cleaved product. These cleaved OPN fragments often exhibit enhanced activity. Specifically, thrombin-induced cleavage unveils a cryptic integrin-binding motif [57] that escalates the activation, proliferation, and migration of hepatic stellate cells, thereby amplifying fibrogenesis [58]. It also releases a C-terminal fragment possessing chemotactic properties for dendritic cells [31].

### 3.2. Animal Models of Prostatic Inflammation

Initially, the correlation of OPN with prostatic inflammation in animal models was delineated using a carrageenan-induced model of chronic prostatic inflammation. Within this model, OPN expression exceeds 2000-fold elevation, concomitant with a significant rise in *Mmp9*, *Col3a1*, and *Col5a2* expression levels [59]. These discoveries led to subsequent evaluations utilizing a bacterial model using transurethral instillation of uropathogenic *E*. *coli*, predominantly inducing inflammation in the dorsal prostate within a week but expanding to the ventral lobe by the two-month mark [51]. The pronounced inflammatory grade acquired in this model culminates in a significant upregulation of OPN at both RNA and protein levels. Investigations employing systemic OPN-KO mice demonstrated that while the acute inflammation severity remains unaltered, the resolution of inflammation and fibrosis is enhanced in the absence of OPN. This is concomitantly linked to improved urinary function, as evidenced by decreased urinary frequency. Moreover, the absence of OPN is correlated with the downregulated expression of multiple genes implicated in extracellular matrix remodeling, fibrosis (such as *Col3a1*, *Dpt*, *Lum*, and *Mmp9*), and inflammation (including *Il16*, *Il1b*, *Cxcl13*, and *Ccl20*) [51]. Similar pro-inflammatory actions of osteopontin have been observed for other disease models. It is well-documented that monocyte/macrophage migration and T-cell Th1 differentiation, activation, and migration are regulated by OPN, and both of these cell types express OPN in inflammatory conditions [60,61]. A study on interstitial pulmonary fibrosis (IPF) indicates that *Spp1* is predominantly expressed in myeloid cells within this condition, and IL-6 augments OPN expression in macrophages, which in turn sensitizes and directs fibroblasts toward other fibrogenic growth factors [62]. Consequently, a plausible primary consequence of OPN deficiency might be the modulation in the distribution and activation of specific immune cells in prostatic inflammatory conditions.

### 3.3. Animal Models of BPH

BPH is a complex pathological entity underscored by marked disruptions in steroid hormone balance. Historically, it has been posited that BPH etiology is predominantly underpinned by androgenic influences. Consequently, the pharmacological intervention employing 5α-reductase inhibitors (5-ARIs) to obstruct the bioconversion of testosterone to the more biologically active 5α-dihydrotestosterone has been a mainstay in efforts to curtail prostate growth. However, a subset of patients exhibit therapeutic resistance, advancing to surgical intervention notwithstanding 5-ARI regimen [63]. Additionally, serum testosterone concentrations diminish with aging, whereas exogenous testosterone administration frequently proves insufficient to elicit BPH in experimental animal models [64]. Conversely, estradiol concentrations within the prostate exhibit relative constancy over the lifespan, resulting in a progressive elevation of the estradiol:testosterone ratio with age [65]. Additionally, the synergistic effect of estradiol and testosterone was postulated to drive BPH pathology [64,66]. Consequently, the mouse model that best emulates human BPH characteristics—including proliferation, fibrosis, and inflammation—is created by subcutaneously administering estradiol and testosterone using slow-release techniques (T+E2 model) [67,68].

By employing RNA-seq in this model, our group discerned that *Spp1* is among the top three genes exhibiting elevated expression in the ventral prostate [68]. Subsequent immunohistochemistry highlighted a substantial upregulation within the prostate lumen and epithelial cells. However, in situ hybridization unveiled that the predominant source of OPN is cells that are found specifically in the lumen, not the epithelial cells. Further morphological analysis, combined with the identification of macrophage markers and lipid staining, ascertained these cells as foam cells (lipid-laden macrophages) manifesting pronounced levels of OPN expression. Additionally, the same cell type was corroborated in human BPH tissue samples, predominantly localizing within the lumen [68]. In the T+E2 model, macrophages are the predominant immune cell subtype during the initial two-week period. While the formation of foam cells is not abrogated in OPN-KO mice, there is a discernible reduction in the total macrophage count within the tissue due to the loss of OPN in OPN-KO mice [68].

Additionally, a genetic model of prostate hyperplasia derived from *Nfib*-knockout mice, which manifests epithelial and stromal hyperplasia alongside augmented collagen deposition, also presents elevated *Spp1* expression; however, the identity of cells producing OPN was not revealed in this study [69].

## 4. Cell-Specific Roles of OPN in the Prostate

**Figure 1:**
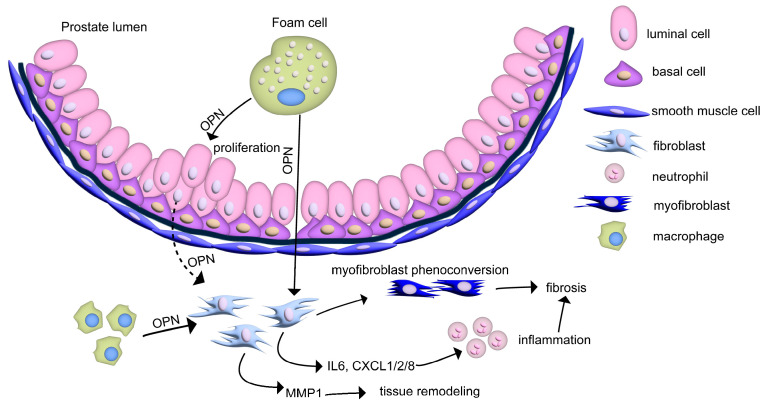
**OPN-mediated cell signaling events suggested in benign prostate pathologies.** Abbreviations/protein names: CXCL1/2/8: chemokine (C-X-C motif) ligand 1/2/8, MMP1: matrix metalloproteinase 1, OPN: osteopontin.

In human BPH prostate samples, OPN demonstrates a heterogenous expression pattern across different cell types at the protein level with evidence of its presence in both epithelial and stromal compartments, including capillaries [55]. However, the task of pinpointing the primary sources of OPN synthesis is complicated by its deposition within the extracellular matrix and its release as a cytokine. In vitro studies have shown that both human epithelial and stromal cells secrete OPN, albeit at minimal levels [55]. Adding to the complexity of understanding the role of OPN, studies using animal models indicate cell-specific upregulation patterns: prostatic OPN expression is heightened exclusively in foamy macrophages in response to steroid hormone imbalance, [68] while bacteria-induced inflammation reveals increased OPN levels within inflammatory infiltrates [51]. OPN is known to be secreted and expressed in a range of immune cells such as macrophages, dendritic cells, neutrophils, eosinophils, NK cells, NKT cells, and T and B lymphocytes. Conversely, the intracellular form of osteopontin (OPN-5) is found in macrophages and dendritic cells, where it promotes pro-inflammatory gene expression and migration [41,70]. This section will delve into the cell-specific roles of OPN, mainly focusing on benign diseases of the prostate. Major findings in the prostate related to OPN signaling and cell-specific roles are summarized in Figure 1.

### 4.1. Resident Prostate Cells

Benign prostate epithelial and stromal cells secrete only low levels of OPN (3–17 pg/mL). However, interleukin-1β (IL-1β) and transforming growth factor-beta 1 (TGF-β1) augment OPN secretion in stromal cells. Meanwhile, only TGF-β1 significantly elevates OPN levels in epithelial cells [55]. Upon exposure to OPN, prostate stromal cells manifest a marked upregulation in the expression of several genes, including *IL6*, *PTGS2*, *CXCL8*, *CXCL1*, *CXCL2*, *ACTA2*, and *MMP1*. The elevated expression of COX2 (PTGS2) and various chemokines indicate that fibroblasts treated with OPN may instigate and amplify inflammatory processes. The upregulation of ACTA2 also implies a potential differentiation towards a myofibroblast phenotype, a cell type associated with profibrotic activities within the prostate [71]. Furthermore, chemokines CXCL8, CXCL1, and CXCL2 have been associated with the recruitment of neutrophils, which subsequently initiate an acute inflammatory cascade [72]. In contrast, the expression of *MMP9* remains unchanged in prostate stromal cells, despite its established association with OPN in various pathologies. Conversely, epithelial cells, when treated with recombinant OPN, do not show marked changes in the aforementioned genes [55]. However, OPN does stimulate the proliferation of epithelial cells [73]. Extrapolating from non-prostatic studies, it is evident that OPN plays a pivotal role in stimulating fibroblast migration and proliferation. Specifically, OPN augments the recruitment of fibroblasts to injury sites, facilitating extracellular matrix deposition [17], and also activates the differentiation of interstitial fibroblasts into myofibroblasts [74]. Cumulatively, these findings underscore the role of OPN in producing a pro-inflammatory milieu and fibrotic alterations in the prostate, along with its contributions to glandular expansion. Nonetheless, these data also suggest that resident prostate cells may not be the primary sources of OPN.

### 4.2. Macrophages and Foam Cells

In the benign prostate milieu, foam cells exhibit the most pronounced expression of OPN [68]. Foam cells are implicated in inciting inflammatory responses and potentiating disease trajectories in conditions such as atherosclerosis [75] and pulmonary fibrosis [76]. In atherosclerosis, the migration and conversion of macrophages to foam cells eventually produce a necrotic core within plaques, ultimately giving rise to atherosclerotic lesions [75]. Various molecular mechanisms underscore the formation of foam cells. For one, they can originate from the uptake of extracellular oxidized lipids [77]. Additionally, hypoxia has been shown to stimulate intrinsic lipid synthesis within macrophages [78]. In the prostate, the overall expression level of fatty acid synthase (*Fasn*) is increased in steroid hormone imbalance, which could spur de novo lipid synthesis, potentially contributing to foam cell development [68].

Recent scientific inquiries have shed light on the intricate relationship between macrophages, lipid regulation, and prostate cancer progression. One notable investigation pinpointed ‘lipid-loaded’ tumor-associated macrophages, marked by high *Spp1* expression, that bore a direct correlation with prostate cancer progression. Furthermore, these macrophages were found to instigate prostate cancer cell migration, mediated through Ccl6 secretion [79]. Another groundbreaking study unveiled cholesterol-rich macrophages in the prostate, revealing that their depletion could attenuate androgen signaling in prostate cancer [80].

Foam cells exhibit an elevated intracellular and extracellular expression and secretion capability of OPN, making them detectable in atherosclerotic plaques using OPN-specific probes [81]. OPN is also elevated in macrophages with heightened lipid metabolism in hepatosteatosis [82]. This indicates that the elevated OPN levels in prostatic foam cells could be a broader phenomenon. While foam cells and OPN were initially thought to have pro-inflammatory roles, current evidence shows that foam cells largely express markers typical of the anti-inflammatory M2 macrophage phenotype, such as *Trem2*, *Cd9*, *Ctsb*, *Fabp4*, and *Lgals3* [83,84,85], and they seem to be more aligned with tissue reparative roles [84,86]. The heightened expression of *Arg1* M2 marker was also found in the prostate steroid hormone imbalance model supporting the predominance of M2 macrophages [68]. Most intriguingly, subsequent investigations discerned that macrophages characterized by elevated *Spp1* expression and diminished *Trem2* expression represent a distinct subset of foam cells. These cells exhibit augmented endoplasmic reticulum stress, increased rates of apoptosis and autophagy, and amplified glycolytic and angiogenic activities, possibly influenced by a hypoxic microenvironment [87], underscoring the inherent heterogeneity within the foam cell population. Moreover, the treatment of oxidized lipids stimulates the expression of pro-inflammatory genes *TNF* and *IL6*, which suggests that foam cells may initially bear an M1 phenotype [88].

Evidence suggests that OPN is present in and modulates the polarization and function of non-foamy macrophages in the prostate. Media derived from prostatic intraepithelial neoplasia stimulate osteopontin expression in RAW 264.7 macrophage cells [89]. Moreover, OPN expression is prevalent in inflammatory infiltrates in the mouse prostate exhibiting macrophage morphology following bacterial infection [51]. Interestingly, the basal tissue number of M1 macrophages is higher in OPN-KO mice compared to wild type mice. However, steroid hormone imbalance increases the M1 macrophage number in wild type mice but not in OPN-KO mice. In contrast, arginase-1 positive M2 cells are also upregulated in OPN-KO mice in this model, although to a lesser extent [68]. These results indicate a shift in M1/M2 ratio driven by the systemic loss of OPN in normal prostate and benign prostate disease.

The literature presents divergent views regarding the pro-inflammatory or anti-inflammatory roles of OPN in macrophages in other tissues. M2 polarization was shown by the conditional knock-in of *Spp1* in myeloid cells, which increased arginase-2 (catalyzes L-arginine to L-ornithine and urea) expression and was protective in nonalcoholic steatohepatitis [82]. A 3D co-culture system for normal epithelial cells and macrophages showed M2 polarization as well as a high level of osteopontin, which stimulated proliferation via Akt and ERK phosphorylation [73]. *Spp1* has been shown to regulate the expression of the PD-L1 M2 polarization gene, and *Spp1* was indicated to inhibit CD4+ T-cell activation [90]. Exogenous OPN added after photothrombotic stroke reduces M1 and increases M2-polarized microglia, the resident macrophage type of the brain [91].

In contrast, lipopolysaccharide (LPS), which drives M1 polarization of macrophages, increases OPN expression [92]. Both the overexpression and recombinant OPN, particularly the phosphorylated form, stimulates cytokines Il-12 and TNFα in M1-differentiated macrophages OPN in peritoneal resident macrophages [20]. OPN also upregulates IL-4, -6, -10, and -1β and TNF-α secretion in M1-differentiated THP-1 cells [93]. Intriguingly, it was discovered that miR-376c-5p inhibits OPN, while a long-non-coding RNA, XIST, targets miR-376c-5p, permitting OPN to drive a pro-inflammatory signature [93]. In a hepatoma model, it was found that OPN indirectly influences macrophages; OPN stimulates the secretion of HMGB1 in HepG2 cells, which in turn induces M1 polarization of monocytes [94]. Systemic OPN-KO also reduces M1 and M2a macrophages but increases M2c subsets [95].

However, some studies remained inconclusive about the impact of OPN on polarization. A study showed that OPN does not affect macrophage polarization but stimulates macrophage survival and migration through the cryptic integrin domain binding to α4 and α9 integrins [96].

The type of response may also depend on the splice variant, specifically, whether or not the intracellular OPN variant (OPN5) is expressed in macrophages. OPN5 has been observed to interact with the (TNFR)-associated factor 3 (TRAF3), safeguarding it from degradation and initiating IRF3 activation, IFN-β synthesis, and the mitigation of virus replication in peritoneal macrophages [97]. Furthermore, OPN-5 cooperates with receptors necessary for antifungal response (dectin-1, TLR2, and mannose receptor) as an adaptor protein to promote phagocytosis and clearance of pneumocystis [98].

Recently, a study analyzing scRNA-seq from 15 varied datasets of human fibrotic diseases identified a unique matrix-associated polarization state of a subset of *SPP1*^+^ macrophages possessing the highest *SPP1* expression level. Such macrophages, proposed to evolve from pro-inflammatory macrophages, expressed genes governing matrix remodeling. However, they are believed to counterbalance the pro-fibrotic activity of other *SPP1*^+^ macrophages by secreting matrix-degrading enzymes [99]. This suggests that *SPP1*^+^ macrophages manifest as a heterogeneous population that either promotes or combats fibrosis, contingent on the disease state, and the polarization state ratio might determine whether inflammation and fibrosis are resolved or persist. Notably, during bacterial-induced prostatic inflammation and consequent fibrosis, osteopontin levels are markedly increased. Fibrosis appears to be mitigated in OPN-KO mice, implying a heightened importance of pro-fibrotic Spp1^+^ macrophages in this model [51].

Pertaining to the general function of OPN, it was observed to induce phagocytosis via the novel receptor αXβ2 (CD11c/CD18) [100]. OPN, in a calcium-dependent manner, binds to bacteria and augments bacterial phagocytosis [100]. CD44 receptor activation triggers macrophage chemotaxis, while the engagement of the β3 integrin influences activation and dispersion [20]. Phosphorylation is also required for interaction with integrins, but not with CD44, as well as for the stimulation of MMP9 in macrophages. OPN from tumor-associated macrophages enhances migration and correlates with unfavorable cancer prognoses [101].

Collectively, these findings underscore the diverse roles of OPN in prostatic macrophages, which might depend on the specific response, be it hormonal, viral, autoimmune, or a reaction to cancer cells. OPN seems to be particularly important in foam cells in the prostate milieu in both benign and malignant conditions [68,79,80]. The role of OPN in macrophages is multifaceted, with evidence suggesting both pro-inflammatory and anti-inflammatory functions, dependent on various factors including specific splice variants and the context of the disease state, providing that more research is needed to fully decipher how OPN contributes to macrophage polarization and function in the prostate.

### 4.3. Other Immune Cells

Beyond macrophages, literature suggests that other immune cells might endogenously express OPN and respond to its secreted form in the prostate. T-cells are the most abundant immune cell type in the prostate: T-cells constitute about 70% of immune cells in BPH, primarily CD4+, but with a significant CD8+ presence [102]. OPN is expressed in T-cells but is also their chemoattractant and co-activator and promotes their longevity and survival [103,104]. Upon activation, T lymphocytes upregulate the expression of the secreted isoform of OPN via the transcription factor T-bet2, which then stimulates the secretion of interleukin-12 from antigen-presenting cells, further promoting T-cell activation [105]. In contrast, macrophage-derived OPN is implied to inhibit CD4+ T-cell activation [90].

Dendritic cells (DCs), professional antigen-presenting cells, are primarily divided into conventional DCs (cDCs) and plasmacytoid DCs (pDCs), with the latter identified as DCs that respond to viral infections through type I interferon secretion [106]. Both cDCs and pDCs are present in normal prostate tissues, but limited data exist about their role in benign disease states [107,108]. In contrast, studies in prostate cancer have emphasized the significance of reduced monocyte-derived DC, cDC, and pDC populations in prostate cancer [107,109,110], indicating the potential anti-tumor effectivity of delivering DCs to promote immune response specific for PCa [111].

OPN exhibits varied functions in the major types of dendritic cells: in pDCs, OPN-5 interacts with the TLR9-IRF7-MyD88 complex to promote IFNα production in pDCs [37,112], while in cDCs, it facilitates the differentiation of IL-17-secreting Th cells by suppressing IL-27 [113].

In conclusion, OPN plays a multifaceted role in modulating immune responses in the prostate, with potentially distinct impacts on T-cells and dendritic cell subsets. The nuanced interactions of OPN, particularly its differential effects on T-cell activation and dendritic cell functionality, highlight the need for further research into studying the immune cell-specific actions of OPN in the prostate.

## 5. Speculating the Role of Luminal OPN in Prostate Disease

In recent studies, notably in the T+E2 model of BPH, there has been a striking observation of elevated levels of OPN within the lumen of prostate ducts, which are anatomically structured to open into the urethra [68]. Immunohistochemical evaluations have provided evidence that epithelial cells are actively endocytosing OPN from the lumen, a process that appears to impact both cellular proliferation and the induction of fibrosis [68]. Notably, even in the context of bacterial inflammation, there is significant luminal staining of OPN, suggesting potential release from a variety of immune cells entering this space [51]. Substances localized within the lumen might exhibit increased functional relevance compared to those tethered within the tissue matrix. For example, certain soluble proteins in the tissue, such as TGF-β1, may experience diffusion challenges due to their confinement within tissue structures [114]. Given the close proximity of epithelial cells to prostatic fluid, proteins present in the lumen are potentially well-situated to regulate a wider spectrum of epithelial cell activities. Moreover, with evidence pointing to the often-compromised integrity of the epithelial barrier in BPH [115], luminal constituents might have enhanced accessibility to the adjacent stroma. A similar interaction between lumen and tissue is evident in the lung, where alveolar macrophages transform into foam cells post exposure to bleomycin, triggering a significant fibrotic reaction [116]. While these findings provide insights into the potential effects of luminally secreted OPN on tissue functions, it remains crucial to conduct additional research to comprehensively understand the underlying mechanisms of this modulation.

## 6. OPN and Prostate Cancer

Prostate cancer, the most common malignancy in men, manifests in a range of phenotypic forms. Prostatic intraepithelial neoplasia is considered a precursor lesion to prostate adenocarcinoma and is characterized by proliferation of atypical cells confined within the prostatic ducts and acini [117]. The Gleason grading system, based on the microscopic appearance, is a vital tool to classify prostate cancers with elevated scores correlating with heightened cancer aggression. This system remains integral in clinical settings to predict prognosis and inform therapeutic decisions [118]. Prostate-specific antigen (PSA) is used as a serum marker for PCa screening, or recurrence after therapy, although it cannot unequivocally discriminate between malignant and benign prostate disease requiring further diagnosis with imaging and/or biopsy [119]. Predominantly, prostate cancer growth is propelled by androgen receptor (AR) signaling. However, infrequently, AR-negative subtypes, such as neuroendocrine and double-negative PCa, develop, representing more aggressive phenotypes [120,121]. Given the pronounced dependency of PCa on androgens, androgen deprivation therapy (ADT) has been the foundational treatment for this malignancy over decades. However, it has been observed that over time, robust resistance mechanisms emerge, allowing the cancer to progress despite androgen withdrawal, culminating in castration-resistant prostate cancer (CRPC) [122]. Typically, prostate cancer metastasizes primarily to the bone, then to distant lymph nodes, the liver, and the thorax [123].

Increased levels of OPN have been detected in various cancers, encompassing bladder, ovarian, lung, kidney, liver, and colorectal cancers, among others [124,125]. It has been demonstrated that OPN can serve as a prognostic marker for breast cancer metastasis [126,127], neuroendocrine neoplasms [128], and melanoma [129]. In various contexts, OPN is implicated in tumor progression by modulating cellular proliferation, angiogenesis, epithelial-to-mesenchymal transition, and immune responses [125].

Several studies have delved into discerning expressional changes across stages of prostate cancer. Notably, osteopontin levels exhibit an increasing trend in cell lines that represent the continuum of prostate cancer progression (from androgen sensitive to CRPC) and correlate with a higher Gleason score, reduced survival [130], and metastatic tendencies [53,131]. The three predominant splice variants (OPNa, OPNb, and OPNc) are markedly upregulated in prostate cancer, particularly in patients with a Gleason grade of 7 or higher [132]. Concurrently, elevated plasma OPN levels were discerned in PCa patients and demonstrate associations with MMP9 and COX2 expression [53]. Some studies have unveiled that osteopontin and PSA (primary marker in clinical use) plasma levels hold comparable efficacy in predicting treatment outcomes in metastatic PCa post radiotherapy [133]. Chemotherapy has also been correlated with increased osteopontin levels in PCa [134].

Pertaining to cellular origin, the bulk of OPN is predominantly expressed in tumor cells in PCa [53]. In genetic PCa models, elevated OPN levels are predominantly detected in epithelial cells, as well as in dispersed cells in the stroma believed to be immune cells [135]. Targeting osteopontin with specific antibodies revealed its role in promoting prostate cancer growth through paracrine/autocrine mechanisms [52]. Intriguingly, in the TRAMP model of PCa (a genetic model that expresses the SV40 T antigen in the prostate), crossing with the systemic knockout OPN model resulted in more aggressive prostate cancer characterized by increased tumor mass, reduced survival, greater dedifferentiation, and a shift towards a neuroendocrine phenotype, which was linked to the downregulation of the TGF-β pathway [136]. This suggests a potential protective role of OPN during the initial phases of prostate carcinogenesis, contrasting with its pro-tumor activity in more advanced stages.

**Figure 2:**
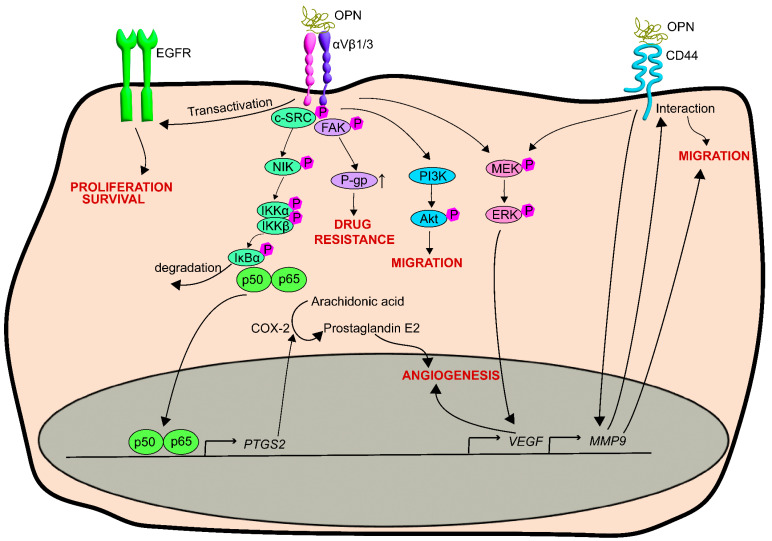
**OPN-mediated cell signaling events identified in prostate cancer cells.** Abbreviations/protein names: COX-2: Cyclooxygenase-2, c-SRC: Proto-oncogene tyrosine-protein kinase Src, EGFR: Epidermal growth factor receptor, ERK: Extracellular signal-regulated kinase, FAK: Focal adhesion kinase, IκBα: NF kappa B inhibitor α, IKKα/β: IkappaB kinase α/β, MEK: Mitogen-activated protein kinase, NIK: NF-κB-inducing kinase, OPN: Osteopontin, p50: Nuclear factor κ-light-chain-enhancer of activated B cells/NFκB, p65: NFκB p65 subunit, P-gp: P-glycoprotein, PI3K: Phosphoinositide 3-kinase.

OPN-mediated signaling pathways in prostate cancer align with those observed in other tumors (Figure 2). Recombinant OPN was found to boost the proliferation of PIN cells through the phosphorylation of Akt and Jun kinase [89]. Through the αvβ3 integrin receptor, OPN stimulates c-SRC phosphorylation, subsequently leading to the phosphorylation of p50/p65 [137]. COX-2, a target protein of this pathway, produces prostaglandin, which drives endothelial cell migration and invasion leading to the stimulation of angiogenesis [137]. Tumor growth is also dependent of COX-2 and is inhibited by COX-2 inhibitor celecoxib and an inhibitor of the EP2 receptor [137].

OPN stimulates CD44 expression and the secretion and activity of MMP9 and promotes the interaction of CD44 and MMP-9, fueling cell migration [138]. This has been associated with heightened gelatinase activity and an increased number of invadopodia [139]. OPN and MMP9 also collaborate to increase the expression and secretion of VEGF, promoting angiogenesis via ERK phosphorylation [140,141]. OPN also induces plasminogen activators, deemed crucial for PCa in forming bone metastases [142,143]. The contribution of OPN to bone metastasis is discussed in detail elsewhere [144].

As a component of the extracellular matrix, OPN can also enhance migration in PC3 PCa cells through the αvβ3 receptor and the stimulation of PI3-kinase activity and AKT phosphorylation [145]. Integrin receptor interactions with the extracellular matrix, including αvβ3 and αvβ1-osteopontin interactions, are vital for activating the EGF receptor and its influence on the proliferation and survival of PCa cells [146,147].

Hypoxia, a common feature in many tumors, arises when rapid proliferation and tumor growth outpace vascular supply [148]. Hypoxia augments OPN expression in PCa cells [134], leading to αvβ3 integrin/FAK-mediated upregulation of the p-glycoprotein drug transporter, thereby heightening resistance to chemotherapeutics both in vitro and in vivo [134,149].

Finally, OPN secreted by PCa cells can reconfigure the immune landscape. OPN is postulated to be a pivotal driver in recruiting myeloid-derived suppressor cells, thereby enhancing prostate cancer survival [150].

## 7. Conclusions

OPN has been unequivocally established as a pivotal molecule in prostatic pathophysiology, manifesting its significance in both benign and neoplastic states of the prostate gland (references are summarized in Table 1). Considering its role in cancer growth and metastasis, fibrosis, and inflammation, pharmacological targeting of OPN may have multiple beneficial therapeutic effects in prostate diseases. However, its discrete cellular mechanisms and the nuanced roles it plays at the cellular level remain an area warranting further investigation. Accumulated evidence underscores the pro-tumorigenic role of OPN in prostate cancer cells, where its augmented expression and secretion ostensibly foster tumor progression and aggressiveness. Contrarily, in benign prostatic conditions, macrophages and foam cells emerge as the primary contributors to OPN secretion. Delineating these specific cellular functions and interactions will be paramount in understanding the therapeutic potential and the molecular intricacies of OPN in prostate health and disease.

## Figures and Tables

**Table 1:** Prostate disease-related publications on OPN.

Prostate Disease	Major Finding	Reference	Citation
**Benign prostate disease**	OPN expression is similar in prostate cancer and BPH.	Thalmann et al., 1999	[52]
	The serum level of OPN is elevated in BPH patients compared to age-matched healthy males.	Castellano et al., 2008	[53]
	The presence of OPN-reactive antibodies is increased in men with BPH vs. healthy men.	Tilli et al., 2011	[54]
	OPN tissue expression is increased with BPH progression (incidental BPH vs. surgical BPH), and OPN induces cytokine expression in prostate stromal cells.	Popovics et al., 2020	[55]
	OPN expression is upregulated in the carrageenan-induced prostatic inflammation mouse model.	Popovics et al., 2018	[59]
	Loss of OPN ameliorates prostatic inflammation and fibrosis in *E*. *coli*-induced mouse model of prostatitis.	Popovics et al., 2021	[51]
	OPN is highly expressed in prostatic luminal foam cells, and the loss of OPN leads to decreased macrophage numbers and improves urinary function.	Popovics et al., 2023	[68]
	*Spp1* expression is elevated in the *Nfib*-knockout prostate hyperplasia model.	Grabowska et al., 2016	[69]
**Prostate cancer**	OPN expression correlates with prostate cancer progression, Gleason score, and reduced survival.	Forootan et al., 2006	[130]
	OPN level correlates with prostate cancer progression and MMP9 expression.	Castellano et al., 2008	[53]
	Plasma osteopontin level is associated with worse survival and metastasis in men with castration-resistant prostate cancer.	Hotte et al., 2002	[131]
	OPN splice variant expression is associated with PCa Gleason grade.	Tilli et al., 2012	[132]
	Osteopontin plasma level predicts treatment outcomes in metastatic PCa post radiotherapy.	Thoms et al., 2012	[133]
	Chemotherapy is associated with increased osteopontin levels in prostate tumors and increased drug transporter expression.	Hsieh et al., 2013	[134]
	OPN level is increased predominantly in epithelial cells, as well as in stromal immune cells, in genetic models.	Khodavirdi et al., 2006	[135]
	OPN-targeting antibody inhibited the growth-stimulatory action of endogenous OPN in Pca cells.	Thalmann et al., 1999	[52]
	TRAMP mice develop more aggressive prostate cancer with a neuroendocrine phenotype when crossed with OPN-KO vs. wild-type mice	Mauri et al., 2016	[136]
	OPN stimulates the phosphorylation of Akt and Jun kinase in PIN cells.	Messex et al., 2022	[89]
	OPN stimulates the c-SRC/p50&p65/COX-2 pathway leading to the production of prostaglandin and the stimulation of angiogenesis.	Jain et al., 2006	[137]
	OPN stimulates the secretion and activity of MMP9 and its interaction with the CD44 receptor, thereby promoting cell migration.	Desai et al., 2007	[138]
	OPN overexpressing prostate cancer cells have increased gelatinase activity and more invadopodia.	Desai et al., 2008	[139]
	OPN and MMP9 cooperate to increase the expression and secretion of VEGF, ERK phosphorylation, and angiogenesis.	Gupta et al., 2013	[140]
	OPN stimulates the phosphorylation of MEK, ERK1/2, and Akt in PCa cells.	Robertson et al., 2010	[141]
	OPN stimulates plasminogen activators in PCa cells.	Angelucci et al., 2002	[142]
	OPN increases the urokinase-type plasminogen activator in PCa cells to promote bone metastasis.	Dong et al., 2008	[143]
	OPN enhances migration in PCa cells via PI3-kinase activity/Akt pathway.	Zheng et al., 2000	[145]
	Integrin receptor–OPN interaction co-stimulate EGF receptor signaling.	Angelucci et al., 2004	[147]
	Hypoxia induces OPN expression in PCa cells.	Hsieh et al., 2013	[134]
	Osteopontin splice variant overexpression enhances resistance to docetaxel.	Nakamura et al., 2016	[149]
	PCa-secreted OPN promotes myeloid suppressor cells.	Brina et al., 2023	[150]
